# Sesamol defends neuronal damage following cerebral ischemia/reperfusion: a crosstalk of autophagy and Notch1/NLRP3 inflammasome signaling

**DOI:** 10.1007/s10787-023-01355-1

**Published:** 2023-10-17

**Authors:** Shorouk Mohamed El-Sayyad, Dina M. Abo El-Ella, Mohamed M. Hafez, Asmaa K. Al-Mokaddem, Bassam Mohamed Ali, Magdy M. Awny, Soad Z. El-Emam

**Affiliations:** 1 Faculty of Pharmacy, Pharmacology and Toxicology Department, October 6 University, Giza, 12585 Egypt; 2 Faculty of Pharmacy, Biochemistry Department, Ahram Canadian University (ACU), Giza, Egypt; 3 Faculty of Veterinary Medicine, Department of Pathology, Cairo University, Giza, 12211 Egypt; 4 Faculty of Pharmacy, Department of Biochemistry, October 6 University, Giza, 12585 Egypt

**Keywords:** Ischemic stroke, Demyelination, Glial fibrillary acidic protein, Connexin43, Inflammasome

## Abstract

**Objective:**

Sesamol (SES) is a phenolic compound found in sesame seed oil. Several studies have revealed its anti-inflammatory and antioxidant properties. However, its complete underlying mechanistic perspective about cerebral ischemia/reperfusion (I/R) lesions has not yet been disclosed. Consequently, we aimed to scrutinize its neuroprotective mechanism against cerebral injury during a global cerebral I/R in a rat model, considering its impact on autophagy and Notch1/NLRP3 inflammasome signaling regulation.

**Methods:**

To affirm our purpose**,** adult Wistar rats were allotted into five groups: sham and the other four groups in which transient global cerebral ischemia was induced by bilateral common ligation (2VO) for 1 h, then reperfusion for either 24 h or 5 days: I/R (1/24), I/R (1/5), SES + I/R (1/24), and SES + I/R (1/5). In treated groups, SES (100 mg/kg, *p.o.*, for 21 days) was administered before cerebral I/R induction. The assessment of histopathological changes in brain tissues, immunohistochemistry, biochemical assays, ELISA, and qRT-PCR were utilized to investigate our hypothesis.

**Results:**

Advantageously, SES halted the structural neuronal damage with lessened demyelination induced by cerebral I/R injury. Restoring oxidant/antioxidant balance was evident by boosting the total antioxidant capacity and waning lipid peroxidation. Furthermore, SES reduced inflammatory and apoptosis markers. Additionally, SES recovered GFAP, Cx43, and autophagy signaling, which in turn switched off the Notch-1/NLRP3 inflammasome trajectory.

**Conclusions:**

Our results revealed the neuroprotective effect of SES against cerebral I/R injury through alleviating injurious events and boosting autophagy, consequently abolishing Notch1/NLRP3 inflammasome signaling.

## Introduction

Cardiac arrest is considered a significant risk factor for global cerebral ischemia, as it leads to the cessation of cerebral blood flow. The brain tissue exhibits a high degree of sensitivity to ischemia, wherein even short periods of ischemia in neurons can trigger a multifaceted cascade of events that may ultimately result in cellular demise (Choudhary et al. 2021). This phenomenon is commonly known as a primary injury. However, the occurrence of secondary injuries arising from cardiac arrest is attributed to the subsequent reperfusion of the brain after resuscitation. This reperfusion leads to the activation of an inflammatory cascade and the production of pro-apoptotic proteins and reactive oxygen species, which in turn cause additional neuronal damage (Sandroni et al. 2021). Cerebral ischemia stimulates the release of glial fibrillary acidic protein (GFAP) by astrocytes, which are the predominant glial cells in the brain (Amalia 2021). GFAP is released to regulate blood flow following ischemia to prevent extension of the infarcted area (Nawashiro et al. 2000). Ischemic damage is exacerbated further after restoring blood reperfusion. Ischemia/reperfusion (I/R) injury alters the structure and function of the blood–brain barrier (BBB) (Genchi et al. 2021). Connexins and pannexins are large-pored non-selective channels that have been shown to play time-dependent roles in the persistence of ischemic injury (Kim et al. 2018). Astrocytic Connexin43 (Cx43) is the most abundant connexin in brain tissue. Under normal conditions, it participates in metabolite exchange between communicated cells by forming gap junction channels or hemichannels, thereby sustaining the CNS environment’s homeostasis (Liang et al. 2020). Under ischemic conditions, Cx43 contributes to the initiation and spread of inflammation by producing pro-inflammatory cytokines, such as tumor necrosis factor-α (TNF-α) and interleukin-1β (IL-1β) (Yu et al. 2020). Inflammasome activation plays a prominent role in the pathogenesis and progression of numerous inflammatory diseases including neurodegenerative diseases and brain injuries (Gao et al. 2017a).

The NOD-like receptor protein 3 (NLRP3) inflammasome is a multi-protein complex inflammatory signaling system that mediates an inflammatory response by releasing inflammatory cytokines as a result of mitochondrial dysfunction (Gong et al. 2018). The NLRP3 inflammasome consists of the NLRP3 receptor, an apoptosis-associated speck-like protein containing a caspase activation recruitment domain (ASC), and pro-caspase1, and its activation in I/R triggers caspase-1 activation, culminating in the release of IL-1 and IL-18 (Latz and Xiao 2013). Moreover, NLRP3 is also activated by the Notch1 signaling pathway (Lee et al. 2020). This pathway is crucial in regulating various cellular processes under physiological conditions such as cell proliferation and differentiation as well as apoptosis. However, Notch1 signaling has a detrimental role in different pathological conditions as it appears to be involved in inflammation and oxidative stress and thus in the progression of cerebrovascular diseases (Cai et al. 2016; Jin et al. 2019). Autophagy, an important catabolic mechanism, maintains cell homeostasis by removing damaged organelles and harmful substances (Mizushima and Komatsu 2011). Studies have shown that autophagy has a pivotal role in maintaining neuronal hemostasis and inhibits NLRP3 inflammasome-mediated inflammation following cerebral ischemia (Luo et al. 2021).

Sesamol (SES), the primary active phenolic component of sesame seed oil, has long been recognized as a health food and used in traditional medicine. Due to its anti-inflammatory and antioxidant effects, SES has a wide range of pharmacological effects (Singh et al. 2020). It exhibits the ability to traverse the blood–brain barrier by virtue of its lipophilic properties. Multiple studies have demonstrated the neuroprotective capability of SES. It is recently found neuroprotective effects against I/R may be attributed to its antioxidative characteristics (Sharma et al. 2020; Wang et al. 2023). Additionally, it can attenuate apoptosis and inflammation in the cerebral I/R injury (Gao et al. 2017b). However, the mechanism by which SES protects against I/R-induced cerebral injury remains unclear. Therefore, in this study, we aimed to explore the mechanism of SES as a neuroprotective agent in global I/R-induced cerebral injury by investigating its impact on autophagy and the Notch-1/NLRP3 signaling pathway.

## Materials and methods

### Drugs and chemicals

Sesamol (SES) was purchased from Sigma-Aldrich (St. Louis, MO, USA). It was dissolved in a saline solution and vigorously mixed using a vortex until full dissolution. SES was administered orally at a dose of 100 mg/kg, chosen based on previous studies (Sharma et al. 2020; Xia et al. 2022). All other compounds were of the highest commercially available purity.

### Animals

In this research, we used adult male Wistar rats aged 10 weeks and weighing 280–300 g. The animals were kept under optimum laboratory conditions (temperature of 24 ± 1 °C) and a 12/12 h dark/light cycle. Before any experimental treatments, rats were allowed to acclimate for one week, allowing free access to standard diet chow and tap water. According to the National Institutes of Health’s Guide for the Care and Use of Laboratory Animals (NIH Publications No. 8023, revised 1978), the Ethics Committee of the Faculty of Pharmacy, October 6 University, approved the study’s design (No. PRE-Ph-2302004).

### Induction of transient global cerebral I/R in rats

Transient global cerebral ischemia was induced by bilateral common ligation (2VO), according to the method mentioned previously by Collino et al. (2006). Briefly, rats were anesthetized with thiopental sodium (30 mg/kg, i.p.), and both common carotid arteries (CCAs) were exposed with a midline longitudinal incision in the neck. CCAs were carefully isolated, and ischemia was initiated by bilateral clamping using nontraumatic artery clamps. After 1 h of occlusion, the clamps were gently removed, and the incision was then stitched, allowing reperfusion for either 24 h or 5 days. The body temperature was adjusted with an overhead heating lamp. Following the surgical procedure, meloxicam (1 mg/kg, S.C.) was administered to relieve pain (Basrai et al. 2016). For the sham group, rats underwent the same surgical procedure, except for CCAs occlusion.

### Experimental design

**Fig. 1:**
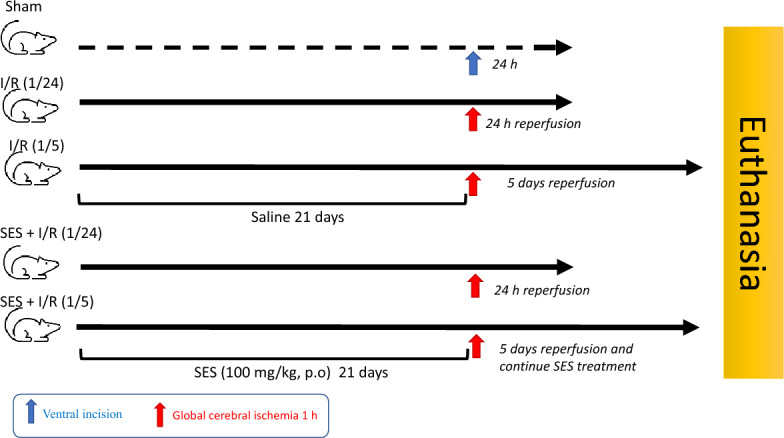
Experimental design flowchart

In this study, sixty-five Wistar rats were randomly allocated into five groups (13 rats/group) as shown in Fig. 1. The groups were as follows:

1. Sham: rats were administered saline (vehicle) orally using gastric gavage for 21 days and then only subjected to ventral incision without clamping the CCAs.
2. I/R (1/24): rats were administered saline (vehicle) for 21 days and then subjected to transient global cerebral ischemia for 1 h, followed by 24 h of reperfusion.
3. I/R (1/5): rats were administered saline (vehicle) for 21 days and then subjected to transient global cerebral ischemia for 1 h, followed by 5 days of reperfusion.
4. SES + I/R (1/24): rats were pretreated with SES orally using gastric gavage (100 mg/kg, *p.o.*) for 21 days and then subjected to transient global cerebral ischemia for 1 h, followed by 24 h of reperfusion.
5. SES + I/R (1/5): rats were pretreated with SES (100 mg/kg, *p.o.*) for 21 days and then subjected to transient global cerebral ischemia for 1 h, followed by 5 days of reperfusion with continuing SES treatment during the perfusion period.

### Samples collection and tissue preparation

Following the end of the reperfusion period, rats were administered thiopental sodium for anesthesia and subsequently euthanized via cervical dislocation. The brains were then immediately removed on ice-cold plates. The samples obtained from all groups were divided into three sets: The first set, comprised of the whole-brain tissues of three rats from each group, was rapidly immersed in 10% formaldehyde to be embedded in paraffin for histological evaluation and immunohistochemistry; the second set, comprising two hippocampi of five rats from each group, was dissected and homogenized in ice-cold PBS supplemented with protease inhibitor for evaluation of biochemical parameters and ELISA measurements; and the third set, consisting of hippocampi from the other five rats, was dissected for total RNA extraction for subsequent quantitative real-time polymerase chain reaction (qPCR) measurement of neuronal gene expression.

### Histopathology

The brains of rats were immediately dissected and kept in 10% neutral buffered formalin for fixation. Each brain was then trimmed in a parasagittal section (longitudinal section lateral to the center line) to expose different regions of interest, including the cerebral cortex, striatum, and hippocampus. A routine tissue processing protocol was followed, and five µm sections were cut and stained with hematoxylin and eosin (H&E) (Bancroft and Gamble 2008). An Olympus BX43 light microscope connected to an Olympus DP27 digital camera was used to examine the tissue and capture images. A quantitative scoring system was applied to assess the detected lesions, including demyelination, neuronal degeneration, and the prominence of endothelial capillaries. These lesions were given a score from 0 to 4 (0 = absent, 1 = mild focal, 2 = mild diffuse, 3 = severe focal, and 4 = severe diffuse lesion), and the mean score for each group was calculated.

### Immunohistochemistry

Tissue sections were sliced into adhesive slides (4–5 µm thick). After rehydration and heat-induced epitope retrieval, the slides were washed, blocked in BSA and hydrogen peroxide, and then incubated overnight at 4 °C in a humid atmosphere with primary mouse anti-rat antibodies for myelin basic protein (MBP; sc-271524, Santa Cruz Biotechnology, Inc., dilution 1:200) or glial fibrillary acidic protein (GFAP; sc-166458, Santa Cruz Biotechnology, Inc., dilution of 1:200). The excess primary antibody was then washed off, and a secondary antibody labeled with HRP (ab97023- Goat anti-mouse HRP labeled antibody, 1:200) was applied for two hours at room temperature, followed by washing. Lastly, the reaction was observed under a light microscope using a DAB-substrate mix (Pierce^™^ DAB Substrate Kit (34,002) from Thermo Scientific, Inc.). Negative control slides were created by omitting the primary antibody. MBP and GFAP expression was quantified as an area percentage of positive expression in 10 microscopic fields to obtain the mean value in each group using CellSens Dimensions software (Olympus, Japan).

### Reverse transcriptase polymerase chain reaction (RT-PCR)

The QIAamp RNeasy Mini Kit (Qiagen, Germany, GmbH) was used to extract RNA from tissue samples. For RT-PCR analysis, primers for the following genes: rat β-actin, NLRP3*,* Notch1, Beclin1, and LC3 were supplied by Metabion (Germany) and are listed in Table 1. QuantiTect SYBR Green PCR Master Mix (Qiagen, Germany, GmbH) was utilized to perform RT-PCR using a Stratagene MX3005P real-time PCR machine. Amplification curves and ct values were determined by the Stratagene MX3005P software. To estimate the variation of gene expression on the RNA of the different samples, the ct of each sample was compared to that of the positive control group according to the “ΔΔct” method stated by Yuan et al., using the following ratio: (2^−∆∆ct^) (Yuan et al. 2006).

**Table 1:** Sequences of oligonucleotide primers for RT-PCR

Target gene	Primers sequences
Rat ß-actin	F: 5’-TCCTCCTGAGCGCAAGTACTCT-3’ R: 5’-GCTCAGTAACAGTCCGCCTAGAA-3’
NLRP3	F: 5’-CAGACCTCCAAGACCACGACTG-3’ R: 5’-CATCCGCAGCCAATGAACAGAG-3’
Notch1	F: 5’-TGGCCTCAATGGATACAAATG-3’ R: 5’-GGGCCAACACCACCTCAC-3’
Beclin-1	F: 5’-TTGGCCAATAAGATGGGTCTGAA-3’ R: 5’-TGTCAGGGACTCCAGATACGAGTG-3’
LC3B	F: 5’-CATGCCGTCCGAGAAGACCT-3’ R: 5’-GATGAGCCGGACATCTTCCACT-3’

### Biochemical evaluation of oxidative stress

Total antioxidant capacity (TAC) and malondialdehyde (MDA) levels were measured in hippocampal samples using commercially available colorimetric test kits (Biodiagnostic, Cairo, Egypt) to assess oxidative stress in tissue homogenates.

### Enzyme-linked immunosorbent assay (ELISA)

The enzyme-linked immunosorbent assay (ELISA) approach was used to estimate Bax, Bcl2, IL-1β, NFκB, and P62 levels in hippocampal tissue homogenates using ELISA kits (MyBioSource, Inc., San Diego, USA, MBS935667); (Cusabio, Houston, USA, CAT. CSB-E13604r); (Cusabio, Houston, USA, CAT. CSB-E08055r); (MyBioSource, Inc., San Diego, USA, CAT MBS722386); and (Creative Diagnostics, USA, CAT. DEIA6457), respectively.

### Statistical analysis

All the data are expressed as the mean ± SD. The difference between groups was statistically analyzed using GraphPad Prism (version 9.5.1, La Jolla, CA, USA). The data were analyzed by one-way analysis of variance (ANOVA), followed by Tukey’s Kramer Multiple Comparison Test. Nonparametric data were analyzed using the Kruskal–Wallis test. A *p* value < 0.05 was considered statistically significant.

## Results

### SES ameliorates histopathological alterations and extensive demyelination induced by global cerebral I/R

**Fig. 2:**
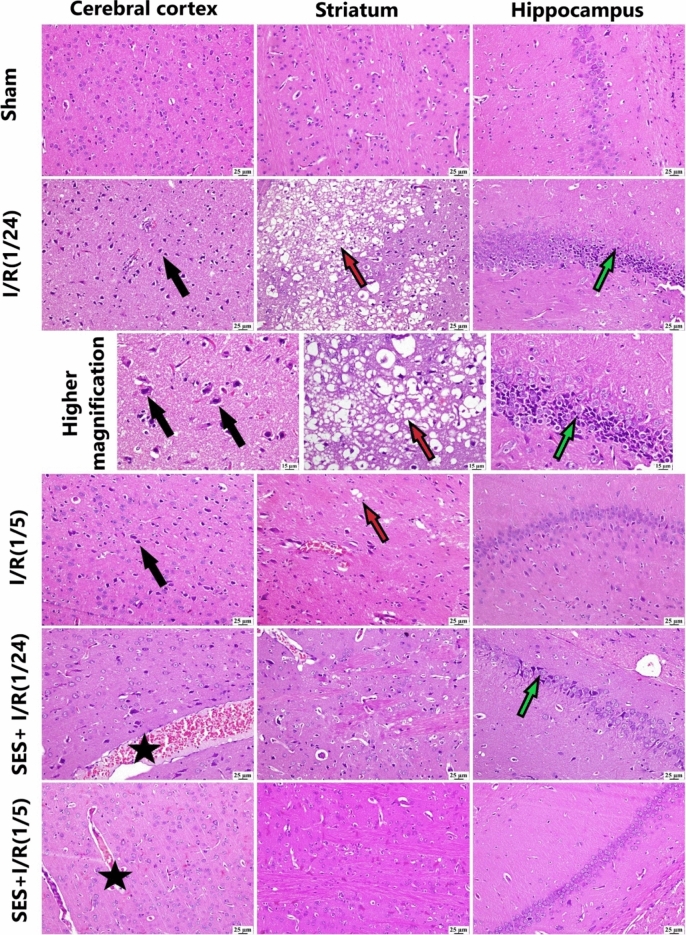
Photomicrographs of the brain stained with H&E: *Sham group* exhibiting normal brain structures, *I/R (1/24) group* displaying a notable presence of dark neurons (black arrows) in the cerebral cortex, extensive demyelination (red arrows), and degenerating neurons (green arrow) in the hippocampus. Higher magnifications of the previous row show dark neurons in the cerebral cortex (black arrows), demyelination (red arrow), and dark degenerating neurons in the hippocampus (green arrow). *I/R (1/5) group* showing dark neurons in the cerebral cortex (black arrows), areas of demyelination (red arrows), some dark neurons in the striatum, and degenerating neurons in the hippocampus, *SES + I/R (1/24) group* showing dilated blood vessels in the cerebral cortex (black star), some degenerating neurons in the striatum, and a few dark neurons (green arrow) in the hippocampus. *SES + I/R (1/5) group* showing dilated blood vessels (black stars) in the cerebral cortex and apparently normal striatum, and hippocampus. Scale bar = 25 µm and 15 µm for higher magnification

The brains of rats from the sham group appeared histologically normal. Histopathological findings are illustrated in Fig. 2. The I/R (1/24) group showed many dark degenerating neurons in the cerebral cortex with neuronophagia and gliosis. The deep cortex exhibited minute focal hemorrhages as well as neuronal edema. Wide areas of infarction were developed, especially in the striatum, which was characterized by neuronal damage and extensive demyelination of nerve fibers. Numerous eosinophilic neurons were observed within the hippocampus. The I/R (1/5) group exhibited neuronal degeneration and neuronophagia as well as focal aggregations of astrocytes in the cerebral cortex. Demyelinated areas were observed in some instances, and dark neurons were frequently detected in the hippocampus. Generally, pre-treatment with SES exerted some protection against the induced brain damage at the two different perfusion times. The SES + I/R (1/24) group showed apparently normal neurons in the cerebral cortex with marked congestion of blood vessels. Minute areas of demyelination and a few dark neurons were observed in the striatum and hippocampus. The SES + I/R (1/5) group exhibited apparently normal cerebral cortex; the striatum showed limited foci of demyelination with distinct and proliferating blood capillaries. The hippocampus was apparently normal.

**Fig. 3:**
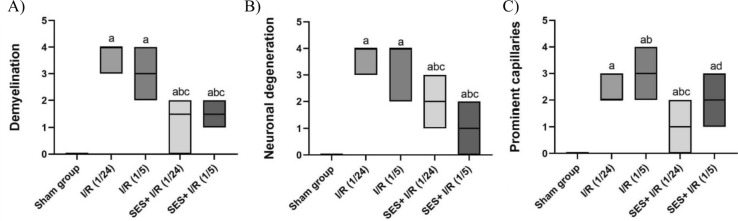
The effect of SES treatment on histological lesion score. **A** Demyelination, **B** neuronal degeneration, and **C** prominent capillaries. The data are presented as median (Max and Min). Significance **a** regarding the control group. Significance **b** regarding I/R (1/24) group. Significance **c** regarding I/R (1/5) group. Significance **d** regarding SES + I/R (1/24) group. Significance: *p* < 0.05

The lesion scores of the identified histological abnormalities are displayed in Fig. 3. In comparison with the sham group, the I/R groups had notably greater lesion ratings. However, SES pre-treatment yielded noteworthy reductions in all assessed histological scores.

**Fig. 4:**
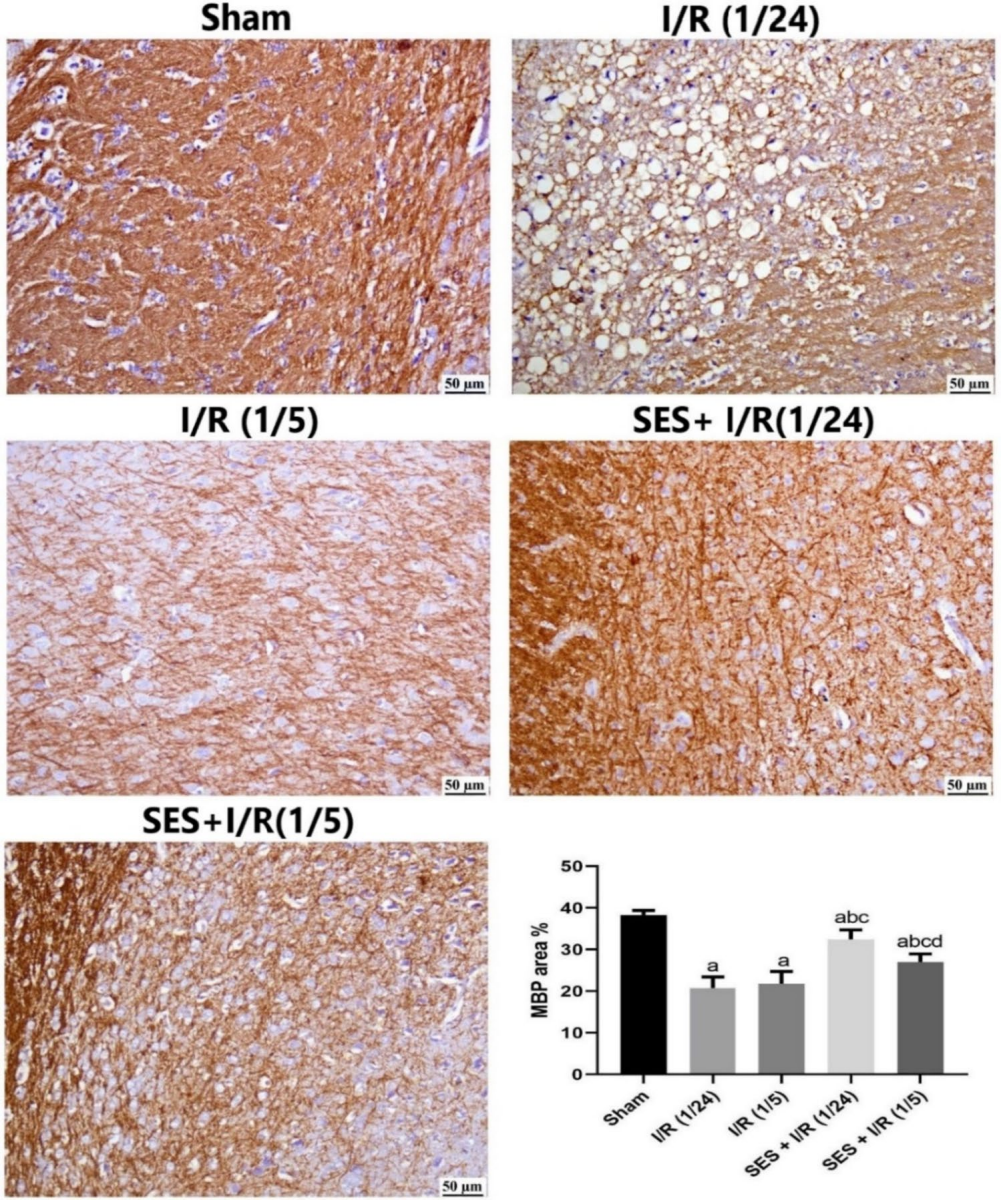
Photomicrographs of the brain’s white matter, illustrating the immune expression of the myelin basic protein (MBP) using immunostaining. *Sham group* showing a normal dense expression of MBP in the white matter, *I/R (1/24) and I/R (1/5)* displaying a marked decrease in MBP expression. *SES + I/R (1/24)* showing an increased positive staining for MBP. *SES + I/R (1/5)* showing a moderate increase in MBP. MBP expression is expressed as area %, and data were presented as means ± SD. Significance **a** regarding the control group. Significance: *p* < 0.05. Significance **b** regarding the I/R (1/24) group. Significance **c** regarding the I/R (1/5) group. Significance **d** regarding SES + I/R (1/24) group. Scale bar = 50µm. *I/R (1/24)* one-hour cerebral ischemia and 24 h reperfusion; *I/R (1/5)* one-hour cerebral ischemia and 5-day reperfusion; and *SES* sesamol

Regarding the immunological expression of MBP as depicted in Fig. 4, it can be observed that the sham group exhibited typical dense myelin in the white matter. Conversely, the I/R (1/24) group displayed a significant reduction in myelin-positive staining, accompanied by the appearance of clear vacuoles. Similarly, myelin loss was also detected in the I/R (1/5) group. Pre-treatment with SES had a protective effect on the white matter, mitigating the occurrence of demyelination generated by ischemia. The expression of MBP was found to be significantly decreased in both the I/R (1/24) and I/R (1/5) groups as compared to the sham group. The pretreated groups exhibited a statistically significant increase in MBP expression as compared to the groups subjected to I/R.

### SES subsides oxidative stress induced by global cerebral I/R injury

**Fig. 5:**
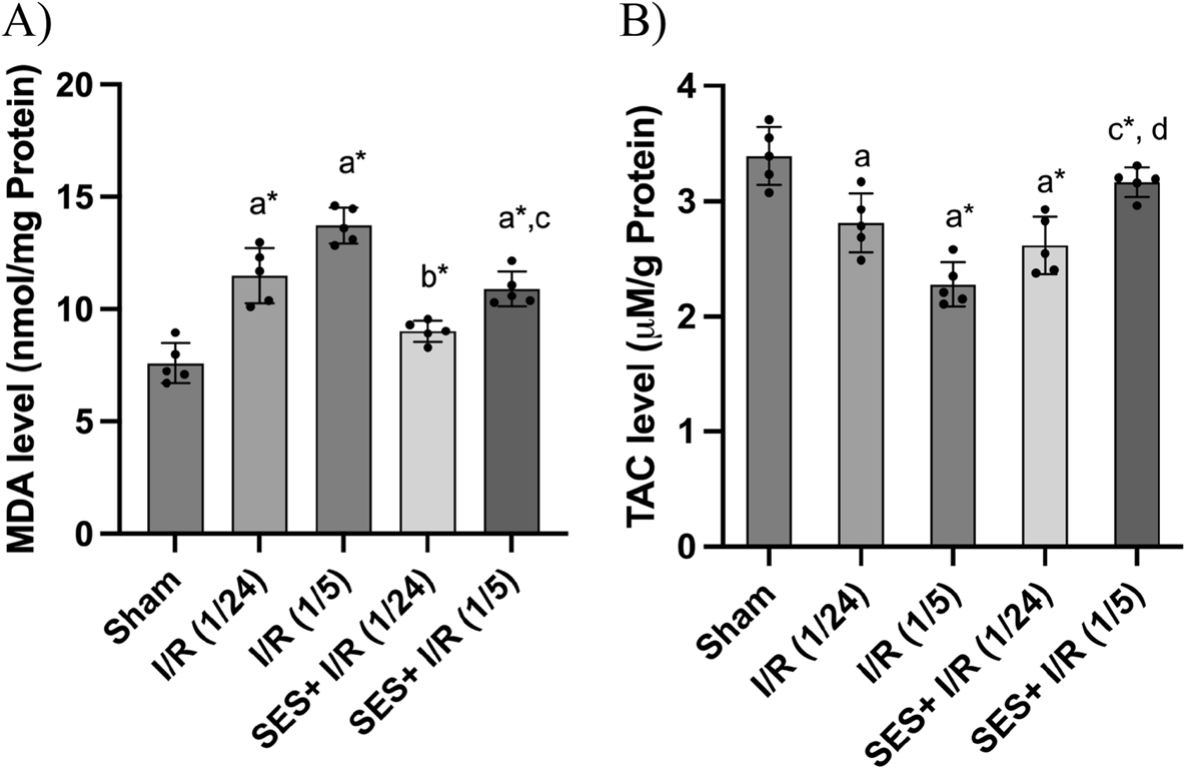
The effect of SES treatment on oxidative stress triggered by global cerebral I/R. **A** MDA levels and **B** TAC levels. The data are presented as mean ± SD (*n* = 5). Significance **a** regarding the control group. Significance **b** regarding I/R (1/24) group. Significance **c** regarding I/R (1/5) group. Significance **d** regarding SES + I/R (1/24) group. Significance: *p* < 0.05, highly significance (*): *p* < 0.001. *I/R (1/24)* one-hour cerebral ischemia and 24 h reperfusion, *I/R (1/5)* one-hour cerebral ischemia and 5-day reperfusion, and *SES* sesamol

The balance of oxidants and antioxidants was used to measure the level of oxidative stress by figuring out how much MDA and TAC were in the brain tissues of all the groups. This balance was deranged in the I/R groups, I/R (1/24) and I/R (1/5), as MDA levels were elevated significantly by 145 and 184%, respectively, as compared with the sham group [(*p* < 0.001), F (4, 20) = 36.69]. To the contrary, TAC levels were reduced to 79.9 and 65.9%, respectively, as compared with the sham group [(*p* < 0.001), F (4, 20) = 19.9]. SES treatment restored the equilibrium between oxidant and antioxidant levels by significantly reducing MDA levels at the two different perfusion times, 24 h and 5 days, as compared with their equivalents in the I/R groups. Additionally, SES pre- and post-treatment after 24 h and 5 days of perfusion increased TAC levels as compared with I/R groups. Furthermore, prolonged treatment with SES for 5 days following reperfusion induced TAC levels significantly higher than those treated for 24 h following reperfusion (*p* < 0.05), as shown in Fig. 5.

### SES represses GFAP expression in global cerebral I/R injury

**Fig. 6:**
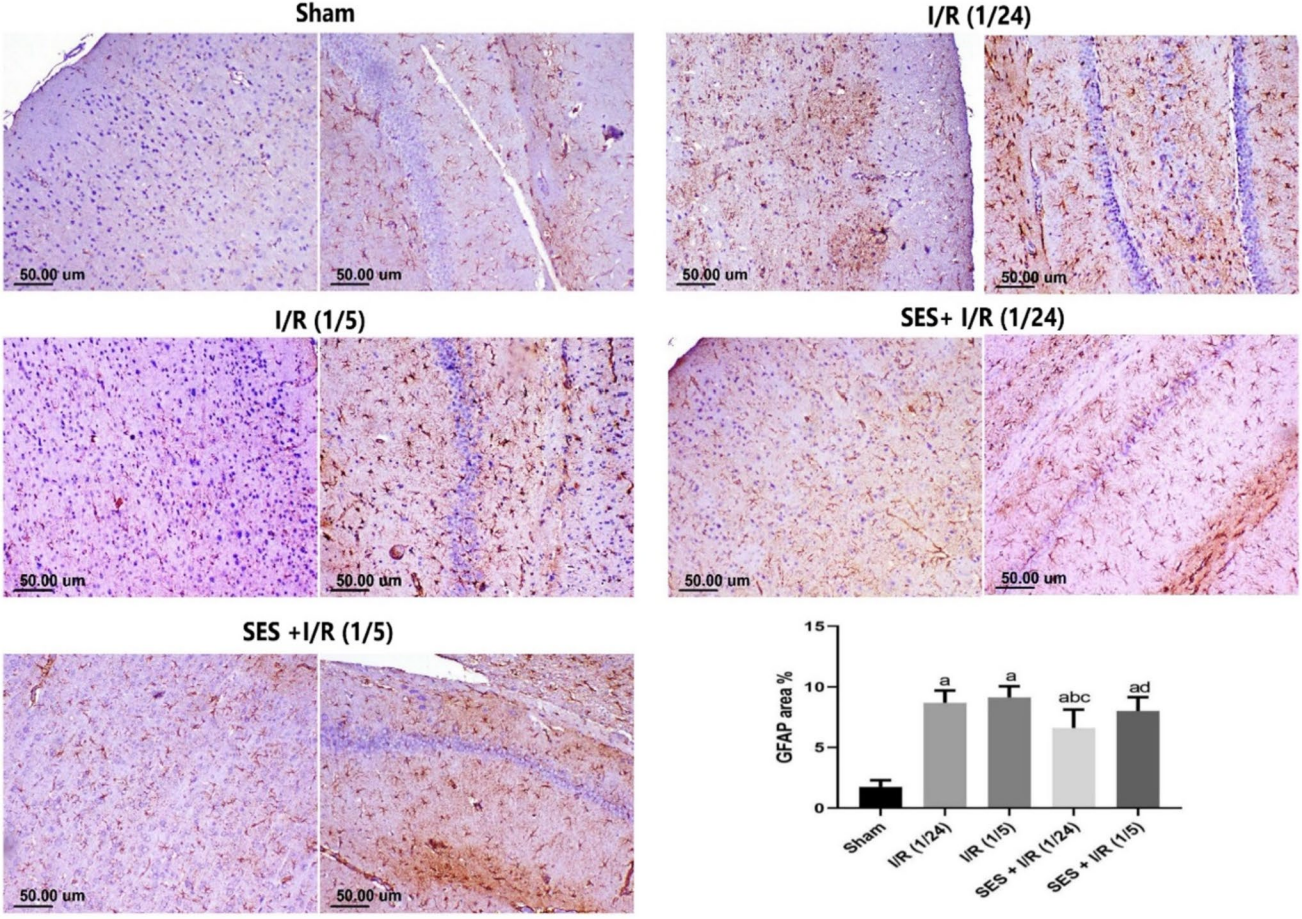
Photomicrographs of brain tissue, with the right image specifically representing the hippocampus and the left image representing the cerebral cortex. These images showcase the immune expression of glial fibrillary acidic protein (GFAP). GFAP is quantified in terms of area percentage. The data are presented in mean ± SD. The significance level at *p* < 0.05. Significance **b** regarding the I/R (1/24) group. Significance **c** regarding the I/R (1/5) group. Significance **d** regarding the SES + I/R (1/24) group. Scale bar = 50µm. *I/R (1/24)* one-hour cerebral ischemia and 24 hours reperfusion; *I/R (1/5)* one-hour cerebral ischemia and 5-day reperfusion; and *SES* sesamol

In terms of GFAP expression, the Sham group exhibited only limited regions with positive staining for GFAP, characterized by minute foci with fine radiating processes. Conversely, the I/R (1/24) group had a significant elevation in GFAP expression. Similarly, the I/R (1/5) group exhibited a notable increase in the number of cells positive for GFAP. The groups that received SES treatment exhibited a reduction in GFAP expression compared to the I/R groups. The SES + I/R (1/24) group had a statistically significant decrease in GFAP positive staining, while the SES + I/R (1/5 group) simply showed a numerical decrease in GFAP positive staining (Fig. 6).

### SES reduces global cerebral I/R-induced inflammatory cascade

**Fig. 7:**
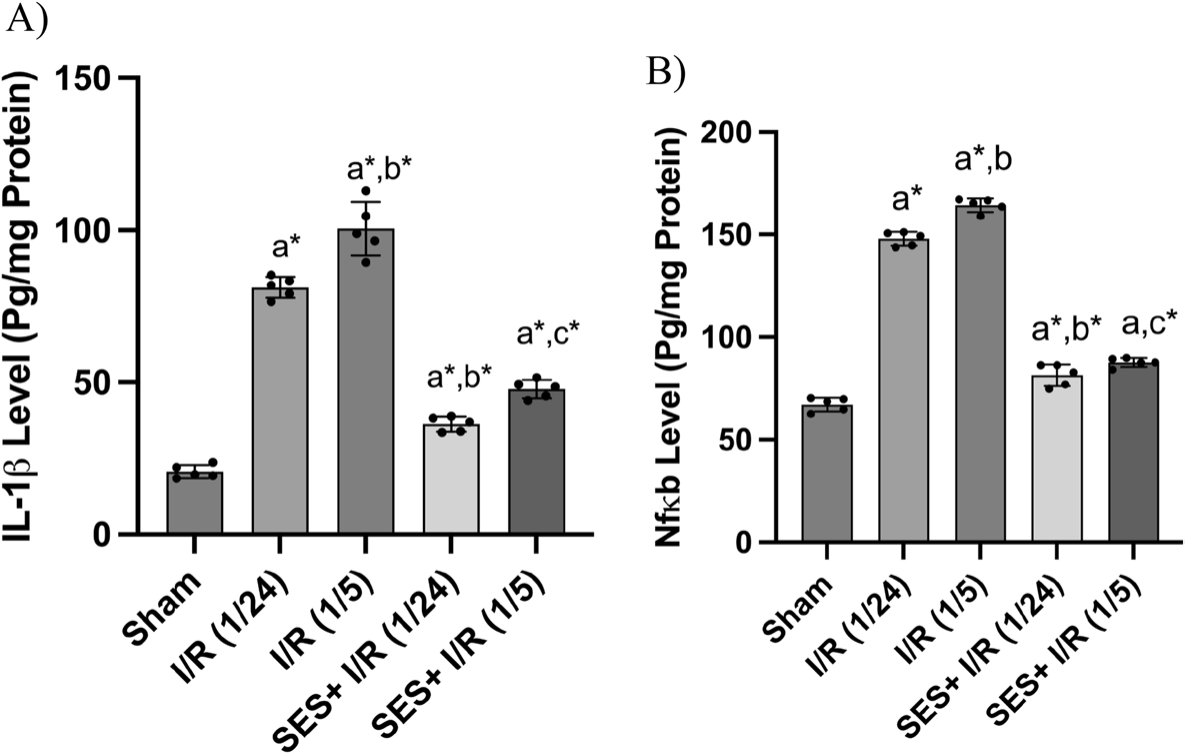
The effect of SES treatment on inflammatory mediators released during global cerebral I/R injury. **A** IL-1β levels and **B** Nfκb levels. The data are presented as mean ± SD (n = 5). Significance **a** regarding the control group. Significance **b** regarding I/R (1/24) group. Significance **c** regarding I/R (1/5) group. Significance **d** regarding SES + I/R (1/24) group. Significance: *p* < 0.05, highly significance (*): *p* < 0.001. *I/R (1/24)* one-hour cerebral ischemia and 24 h reperfusion, *I/R (1/5)* one-hour cerebral ischemia and 5-day reperfusion, and *SES* sesamol

The results of the one-way ANOVA indicated statistically significant differences among the groups in terms of the levels of IL-1β [F (4, 20) = 246.2, *p* < 0.001] and Nfκb [F (4, 20) = 711.5, *p* < 0.001]. The inflammatory cascade was activated in brain tissues due to global cerebral I/R injury in a time-dependent manner regarding reperfusion (Fig. 7). The levels of Nfκb and IL-1β were increased by 221% and 391%, respectively, after 24 h of reperfusion. After 5 days of reperfusion, Nfκb and IL-1β levels were further increased by 242% and 483%, respectively, as compared to the sham group (*p* < 0.001). However, pre- and post-treatment with SES (100 mg/kg, *o.p.*) significantly suppressed the levels of these inflammatory mediators as compared to the I/R groups (*p* < 0.001).

### SES halts apoptosis triggered by global cerebral I/R

**Fig. 8:**
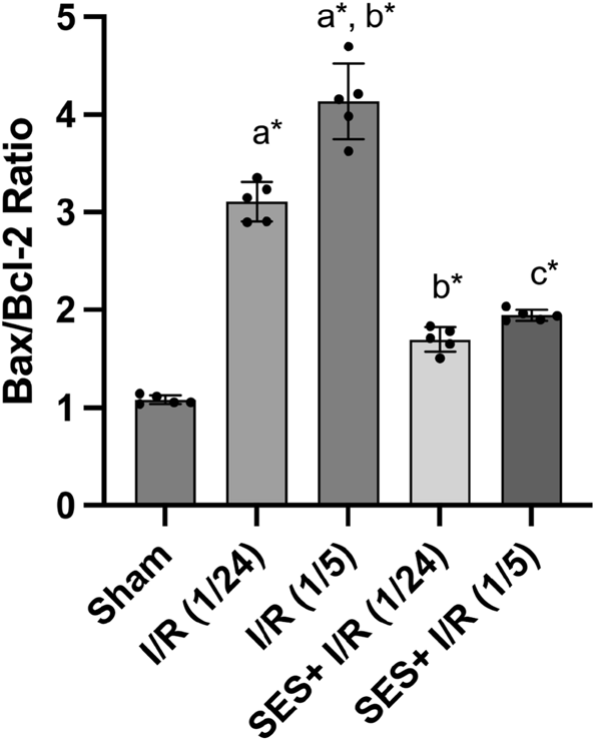
The effect of SES treatment on Bax/Bcl2 ratio after global cerebral I/R injury. The data are presented as mean ± SD (*n* = 5). Significance **a** regarding the control group. Significance **b** regarding I/R (1/24) group. Significance **c** regarding I/R (1/5) group. Significance **d** regarding SES + I/R (1/24) group. Significance: *p* < 0.05, highly significance (*): *p* < 0.001. *I/R (1/24)* one-hour cerebral ischemia and 24 h reperfusion, *I/R (1/5)* one-hour cerebral ischemia and 5-day reperfusion, and *SES* sesamol

The determination of the BAX/Bcl-2 ratio was conducted to assess the level of apoptosis resulting from global cerebral I/R injury. A significant alteration in the Bax/Bcl-2 ratio was observed among the groups as indicated by the results of a one-way ANOVA F (4, 20) = 174.2, *p* < 0.001]. The Bax/Bcl-2 ratio exhibited a substantial increase in proportion to the timing of reperfusion, namely in the I/R (1/24) and I/R (1/5) groups, as compared to the sham group (*p* < 0.001). Pre- and post-treatment with SES in global cerebral I/R resulted in a substantial reduction in apoptosis as evidenced by a decreased Bax/Bcl-2 ratio, compared to the I/R groups (Fig. 8).

### SES improves the gap junction protein, Cx43, level and boosts autophagy after global cerebral I/R

**Fig. 9:**
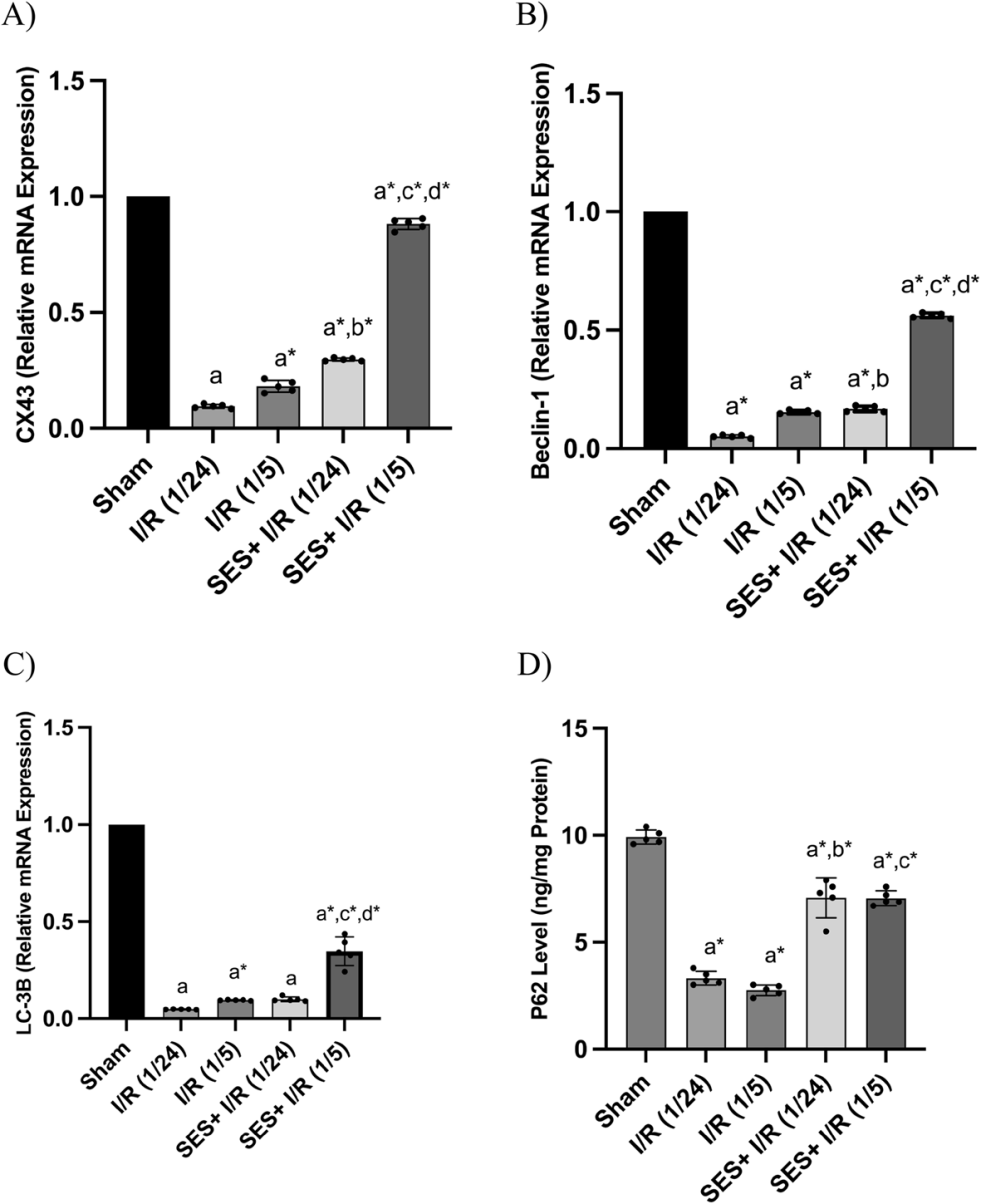
The impact of SES treatment on the degradation of Cx43 and the suppression of autophagy caused by global cerebral I/R injury. **A** Cx43 relative mRNA expression, **B** Beclin-1 relative mRNA expression, **C** LC-3B relative mRNA expression, and **D** P62 level in ng/mg protein. The data are presented as mean ± SD (*n* = 5). Significance **a** regarding the control group. Significance **b** regarding I/R (1/24) group. Significance **c** regarding I/R (1/5) group. Significance **d** regarding SES + I/R (1/24) group. Significance: *p* < 0.05, highly significance (*): *p* < 0.001. *I/R (1/24)* one-hour cerebral ischemia and 24 h reperfusion, *I/R (1/5)* one-hour cerebral ischemia and 5-day reperfusion, and *SES* sesamol

The findings from the one-way ANOVA revealed significant variations across the groups in relation to the cerebral expression levels of Cx43 [F (4, 20) = 3421, *p* < 0.001], Beclin-1 [F (4, 20) = 11,198, *p* < 0.001], and LC-3B [F (4, 20) = 715.8, *p* < 0.001]. The statistical analysis also revealed significant differences in the protein levels of P62 [F (4, 20) = 174, *p* < 0.001]. The mRNA expression of Cx43 in hippocampal astrocytes was notably downregulated by 90.45% and 81.8% in the I/R (1/24) and I/R (1/5) groups, respectively, following global cerebral I/R. This downregulation of Cx43 mRNA expression had subsequent effects on neuronal autophagy. The proteins Beclin-1, LC-3B, and P62 are widely acknowledged indicators of the level of autophagy. In this study, we assessed the level of autophagy in the context of global cerebral ischemia at two distinct time points of perfusion, namely 24 h and 5 days. Additionally, we examined the impact of SES therapy on the regulation of Cx43 and autophagy by measuring the mRNA expression of Beclin-1 and LC-3B, as well as the protein expression of P62, using the ELISA technique. In both reperfusion timings, the mRNA expression of Beclin-1 and LC-3B exhibited a considerable decrease, whereas the protein level of P62 was notably decreased in the I/R groups. In contrast, the administration of SES resulted in a significant upregulation of mRNA levels for Cx43, Beclin-1, and LC-3B, particularly when the reperfusion period was extended to a duration of 5 days. Similarly, the administration of SES resulted in a notable increase in the level of P62 protein when compared to the I/R groups (*p* < 0.001) (Fig. 9).

### SES reduces Notch1/NLRP3 inflammasome signaling following global cerebral ischemia

**Fig. 10:**
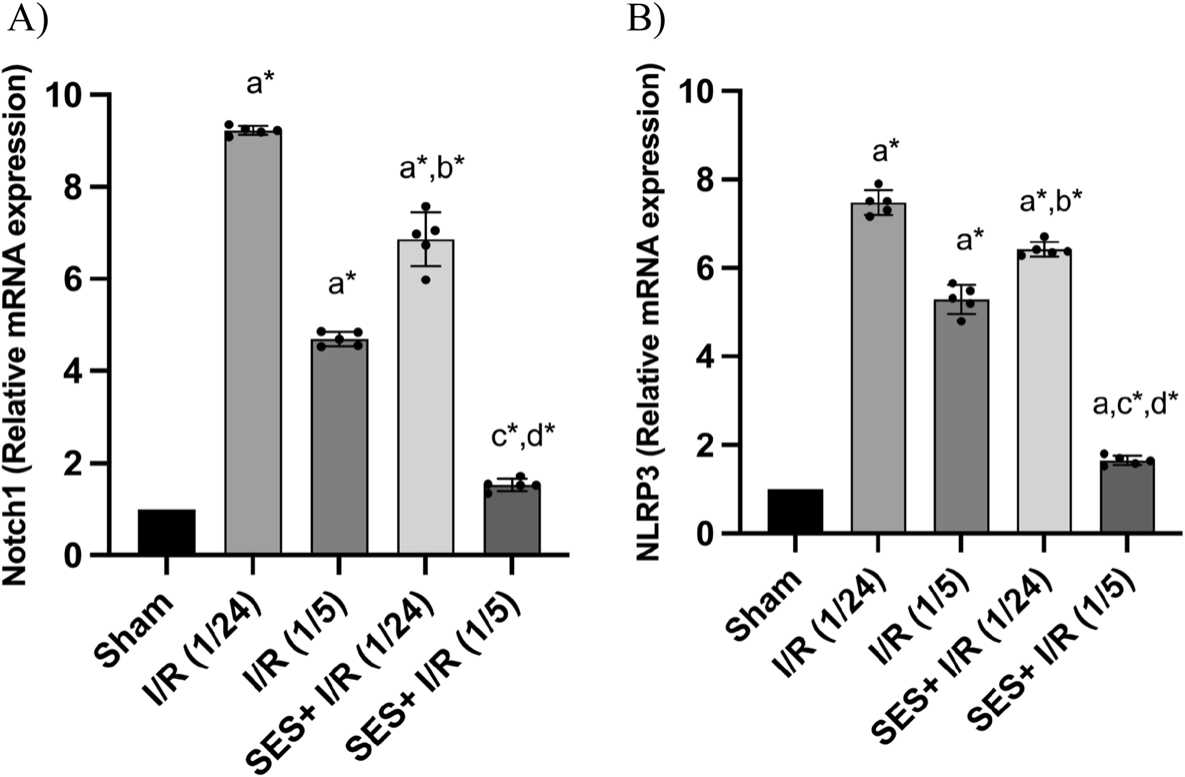
The effect of SES intervention on the expression of Notch1 and NLRP3 genes in the context of global cerebral I/R injury. **A** Notch1 relative mRNA expression and **B** NLRP3 relative mRNA expression. The data are presented as mean ± SD (*n* = 5). Significance **a** regarding the control group. Significance **b** regarding I/R (1/24) group. Significance **c** regarding I/R (1/5) group. Significance **d** regarding SES + I/R (1/24) group. Significance: *p* < 0.05, highly significance (*): *p* < 0.001. *I/R (1/24)* one-hour cerebral ischemia and 24 h reperfusion, *I/R (1/5)* one-hour cerebral ischemia and 5-day reperfusion, and *SES* sesamol

The results of the one-way ANOVA indicated significant differences among the groups in terms of the cerebral mRNA expression levels of Notch1 [F (4, 20) = 789.2, *p* < 0.001] and NLRP3 [F (4, 20) = 931, *p* < 0.001]. The activation of Notch-1/NLRP3 signaling was seen in response to widespread cerebral ischemia. At both reperfusion time points, I/R significantly increased the relative gene expression levels of Notch1 and NLRP3, with a greater rise observed after 24 h of reperfusion. The administration of SES therapy resulted in a notable reduction in the upregulation of Notch1 gene expression (by 26.7% and 68.1%) and NLRP3 gene expression (by 15.72% and 69%) compared to the I/R (1/24) and I/R (1/5) conditions, respectively (*p* < 0.001) (Fig. 10).

## Discussion

The effectiveness of SES has been confirmed in several experimental studies as a therapeutic agent in several contexts, including its anticancer properties (Liu et al. 2017), anti-aging (Ren et al. 2020), renal protective (Singh et al. 2020), hepatoprotective, and antihyperlipidemic agents (Xie et al. 2021). In this study, we used a rat model of global cerebral I/R induced by bilateral common ligation (2VO) to evaluate the neuroprotective effects of SES, shedding light on its anti-inflammatory mechanism and its role in regulating autophagy and Notch1/NLRP3 inflammasome signaling. In the present investigation, it was observed that global cerebral I/R led to the occurrence of oxidative stress at both reperfusion durations, namely 24 h and 5 days. Mitochondrial dysfunction and oxidative stress are critical elements that contribute to the exacerbation of severe damage induced by cerebral I/R (Wu et al. 2020). Numerous researchers have made significant advancements in the field of cerebrovascular illnesses over the past ten years and come to the conclusion that the NLRP3 inflammasome and its downstream inflammatory components are involved in causing severe cerebral I/R injury (Luo et al. 2021). Brain neurons are most sensitive to cerebral blood flow hypoperfusion, resulting in neuronal injury in different regions of the brain owing to the development of the inflammatory detriment mediated by NLRP3 after global cerebral I/R (Franke et al. 2021). Cerebral I/R injury was determined by the changes in histopathological findings, which revealed neuronal degeneration and demyelination implied by repressed MBP. This is consistent with earlier findings demonstrating that one of the mechanisms causing white matter damage following injury after whole-brain ischemia is oligodendrocyte death-induced demyelination (Chen et al. 2013; Zhao et al. 2014). This was described by the study of Du et al., since microglia activation and prominent neuroinflammation following global cerebral ischemia in animal models contribute to demyelination (Du et al. 2020). Our study is congruent with several studies evidencing that enormous ROS production, increased GFAP expression, and apoptosis signaling are the main cornerstones in cerebral I/R-induced brain injury (Yuan et al. 2020; Campanile et al. 2022; Du et al. 2023; Hu et al. 2023) because they have a direct influence on halting autophagy, which has a significant role in neuroprotection (Carloni and Balduini 2020). Different studies have reported the impact of autophagy on regulating Notch1 signaling. Autophagy mediates Notch1 receptor degradation by being captured by pre-autophagosome vesicles (Casares-Crespo et al. 2018; Chen et al. 2021). In turn, activation of Notch1 signaling exacerbates I/R-induced inflammatory injury by means of an upregulated NLRP3 inflammasome (Jin et al. 2020). Additionally, several studies have shown that Notch signaling is upregulated after cerebral ischemia injury in an animal model (Yang et al. 2012; Ren et al. 2018). Therefore, the present study investigated the impact of SES on the autophagy/Notch1/NLRP3 axis in cerebral I/R-induced injury. A growing body of evidence has demonstrated that the neuroprotective effect of SES and reduction in infarction size during I/R are owing to its antioxidant, anti-inflammatory, and anti-apoptotic properties (Gao et al. 2017c; Sharma et al. 2020). However, its effect on autophagy, Notch1, and NLRP3 inflammasome signals is still unclear. In line with previous studies, our results demonstrated the neuroprotective effect of SES against cerebral I/R-driven injury. The neuronal damage was more pronounced after 5 days than after 24 h of reperfusion after ischemia, since restoring blood flow may result in secondary injuries in addition to those induced during ischemia, which can eventually lead to structural and functional impairment of brain tissues (Vongsfak et al. 2021; Zeng et al. 2022). However, SES treatment ameliorated the neuronal damage in both durations of reperfusion by restoring the myelin basic protein. SES can reduce the pro-inflammatory response and abate microglia-mediated inflammation, as revealed in different models (Keowkase et al. 2018; Wu et al. 2023). SES exerts an anti-apoptotic effect, as reported by Feng and co-authors, who found that SES can diminish caspase-3 and Bax levels and boost Bcl2 after spinal cord injury in a mouse model (Keowkase et al. 2018). The degree of apoptosis and inflammatory response play a significant role in the extent of neuronal damage after I/R. BAX and BAK proteins are exposed to oligomerization on the mitochondrial outer membrane in response to apoptotic activation; thus, obstructing this process by Bcl2 protein can prevent mitochondrial permeabilization and hence preclude cell death (Keowkase et al. 2018). Inflammation and excessive cell death during cerebral I/R insults culminate in an imbalance between apoptosis and autophagy that can aggravate further neuronal damage (Luo et al. 2021). Bax-induced apoptosis decreased autophagy by lowering Beclin-1 (Lu et al. 2019). Appropriate autophagy may result in neuroprotection after acute brain injury, such as cerebral I/R, unlike excessive autophagy, which may accelerate cell death (Nabavi et al. 2019; Mo et al. 2020; Luo et al. 2021). Ample studies have demonstrated that enhancing intracellular degradation by autophagy alleviates the inflammatory response and apoptosis provoked by I/R injury (Sun et al. 2020; Luo et al. 2021; He et al. 2023). In accordance with these studies, we found that SES enhanced autophagic activity in cerebral I/R injury, and much improvement was observed when SES was administered during the five days of reperfusion, as evidenced by upregulated Beclin-1 and LC3B mRNA expression and increased P62 levels. In addition, SES halted Cx43 degradation to exert neuroprotective effects since Cx43 correlates directly with inflammasome signaling and since Cx43 hemichannels mediate the release of ATP, which is an essential activator for inflammasome signaling (Mugisho et al. 2019). Additionally, as revealed by Wang et al., the suppression of Cx43 degradation enhanced the switch of astrocytes from a pro-inflammatory to an anti-inflammatory state (Wang et al. 2020). Furthermore, SES treatment suppressed mRNA Notch1 as well as mRNA NLRP3 expression, particularly after 5 days of reperfusion, since boosting autophagy can mediate the degradation of Notch1, which has a significant role in triggering NLRP3 activation via autophagosomes (Wu et al. 2016). Similarly, Lee and the co-authors stated in their study on keloid fibroblasts that rapamycin-induced autophagy substantially decreased Notch1 and NLRP3 inflammasome protein expression (Lee et al. 2020).

## Conclusion

It can be inferred that SES plays a beneficial role in mitigating cerebral I/R injury through the reduction of oxidative stress and apoptosis. This leads to the activation of autophagy and the regulation of Notch1/NLRP3 signaling pathways. SES demonstrates potential as an effective drug for providing neuronal protection against global cerebral I/R injury and cerebrovascular diseases. Moreover, we propose doing a more extensive examination of the effects of SES treatment on the activation of astrocytes in the context of neuroinflammatory illnesses, which should be considered for future research endeavors.

## Data Availability

No additional data are available.
